# NK Cells Are Not Required for Spontaneous Autoimmune Diabetes in NOD Mice

**DOI:** 10.1371/journal.pone.0036011

**Published:** 2012-04-25

**Authors:** Joshua N. Beilke, Craig T. Meagher, Karoline Hosiawa, Marine Champsaur, Jeffrey A. Bluestone, Lewis L. Lanier

**Affiliations:** 1 Department of Microbiology and Immunology, University of California San Francisco, San Francisco, California, United States of America; 2 Cancer Research Institute, University of California San Francisco, San Francisco, California, United States of America; 3 Proctor Foundation, University of California San Francisco, San Francisco, California, United States of America; 4 Division of Rheumatology, Department of Medicine, University of California San Francisco, San Francisco, California, United States of America; 5 Diabetes Center, University of California San Francisco, San Francisco, California, United States of America; University Hospital of Heidelberg, Germany

## Abstract

NK cells have been shown to either promote or protect from autoimmune diseases. Several studies have examined the role of receptors preferentially expressed by NK cells in the spontaneous disease of NOD mice or the direct role of NK cells in acute induced disease models of diabetes. Yet, the role of NK cells in spontaneous diabetes has not been directly addressed. Here, we used the NOD.NK1.1 congenic mouse model to examine the role of NK cells in spontaneous diabetes. Significant numbers of NK cells were only seen in the pancreas of mice with disease. Pancreatic NK cells displayed an activated surface phenotype and proliferated more than NK cells from other tissues in the diseased mice. Nonetheless, depletion of NK cells had no effect on dendritic cell maturation or T cell proliferation. In spontaneous disease, the deletion of NK cells had no significant impact on disease onset. NK cells were also not required to promote disease induced by adoptively transferred pathogenic CD4^+^ T cells. Thus, NK cells are not required for spontaneous autoimmune diabetes in NOD mice.

## Introduction

The primary role of Natural Killer (NK) cells in the immune system is to police the body for cells that have been infected by pathogens or become tumorigenic [1]. Upon encountering these targets, NK cells engage in an assortment of receptor - ligand interactions and destroy cells that elicit sufficient activating signals, countering inhibitory signals, to promote the stimulation of NK cells. Many of the activating receptors expressed by NK cells recognize self-ligands; thus, by nature, NK cells are autoreactive. While once thought of as being important only for innate immunity, recent studies have clearly demonstrated that NK cells share characteristics with antigen-specific T cells [2], [3], [4]. The ability of NK cells to rapidly elicit robust immune responses, in combination with their ‘self-reactive’ nature, make them potential players in autoimmune diseases.

Prior studies have demonstrated that NK cells can either protect against or promote autoimmunity [5], [6]. In some models of experimental autoimmune encephalomyelitis (EAE), NK cells reduce the magnitude of the T cell response by directly killing activated T cells [7], [8]. In this case, improving the function of NK cells by blocking inhibitory signals delivered via NKG2A reduced the severity of EAE. Additionally, NK cells at the site of inflammation in the spinal cord could prevent T cell activity and reduce EAE severity by the secretion of interferon gamma (IFNγ) [9]; however, if IL-18 was given during the induction of EAE, NK cells promoted disease [10]. Likewise, NK cells can have diametrical roles in rheumatoid arthritis; one study demonstrated that the depletion of NK cells resulted in more severe arthritis with the authors suggesting that NK cells inhibited Th17 cells by producing IFNγ [11], whereas another study showed that the depletion of NK cells reduced the severity of bone erosion, demonstrating *in vitro* that NK cells promoted osteoclast differentiation [12].

Similarly, in animal models of autoimmune diabetes, NK cells can either be protective or harmful. For example, in NOD mice, which develop diabetes spontaneously, the activation of NK cells with poly I:C protects against diabetes through the secretion of IFNγ [13]. Yet, NK cells were also found to be disease-promoting in NOD mice in models where disease was accelerated by CTLA4-Ig treatment [14] or in BDC2.5 NOD mice, whose CD4^+^ T cells express a transgene for an antigen-specific TCR to islets [15]. Further evidence that NK cells might promote disease in NOD mice are the examples that blocking the activating receptors NKG2D [16] or NKp46 [17], which are highly expressed by NK cells, dramatically reduced the onset of disease. Another study has shown that NK cells in NOD mice infiltrate the pancreas and appear to be activated, leading the authors to conclude that NK cells promote diabetes [18]. Thus, recent evidence suggests that NK cells promote the onset of diabetes in the NOD mouse in some model systems; however, none of these studies directly examined the requirement of NK cells in the onset of spontaneous disease. Herein, using a congenic model of the NOD mouse that allows direct targeting of NK cells, we examined the role of NK cells in the spontaneous onset of autoimmune diabetes.

## Materials and Methods

### Mice

Dr. Albert Bendelac (Univ. Chicago) kindly provided NOD.B6-(*D6Mit254*-*D6Mit289*)/CarJ (referred to as NOD.NK1.1) mice. NOD/MrkTac and NOD/MrkBomTac-*Prkdc^scid^* (referred to as NOD.*Scid*) mice were purchased from Taconic. Dr. Mark Anderson (UCSF) provided B6.H2^g7^ mice. Dr. Jeffrey Bluestone (UCSF) generated the NOD.Cg-*Prkdc^scid^ Il2rg^tm1Sug^*/JicTac mice (referred to as NSG). *Rag2^−/−^* NOD.NK1.1 mice were generated by breeding NOD.NK1.1 mice by NOD.*Rag2^−/−^* mice. All mice were maintained in the specific pathogen-free animal facility of the University of California, San Francisco. The Institutional Animal Care and Use Committee of the University of California, San Francisco approved animal protocols.

### Pancreatic digestions

Lethally anesthetized mice were perfused with 30 ml of a 2% heparin PBS solution. The pancreas was then placed in 2 ml of media containing 2 mg/ml of collagenase type V (Sigma) and 10 µg/ml DNAse I (Sigma) for 30 min at 37°C. Homogenates were then resuspended in a 40% Percoll (in PBS) gradient layered over a 60% Percoll (in PBS) layer and were centrifuged for 20 min at 300×g. The buffy layer was then removed and washed to prepare the cells for flow cytometry or *in vitro* functional assays.

### Islet isolation

Pancreata were perfused with collagenase type V (Sigma-Aldrich) and digested for 12 min in a 37°C water bath. Islets were purified by using Histopaque and handpicked. Purified islets were dissociated by incubation with an enzyme-free dissociation buffer (Invitrogen) and mixing by pipette.

### NK cell stimulation

NK cells from spleen were enriched by incubating splenocytes at 4°C for 15 minutes with anti–glycophorin A (Ter119), anti-CD4 (GK1.5), anti-CD5 (53-7.3), anti-CD8 (YTS 169.4), and anti-CD19 (1D3), washed, and then resuspended with metallic beads coated with goat anti–rat IgG (QIAGEN) for 30 minutes at 4°C. Antibody-labeled cells were removed by magnetic separation. Enriched NK cells were incubated on plates coated with 10 µg/mL anti-NKG2D (CX5), anti-Ly49D (4E5) (BD Biosciences), or an isotype-matched control mAb (blockade with 2.4G2 had no effect on the stimulation, excluding activation via CD16 on NK cells) or with 20 ng/mL IL-12 and 5 ng/mL IL-18 (R&D Systems) in the presence of brefeldin A. Cells were surface stained with mAbs to NKp46 (R&D Systems), NK1.1, T cell receptor β (TCRβ) (H57-597) (BioLegend), and CD107a (1D4B) (BD Biosciences), and then permeabilized and stained intracellularly with anti-IFNγ mAb (XMG1.2) by using an Intracellular Staining Kit (BD Biosciences).

### Multicolor immunofluorescent labeling and confocal microscopy

Seven-micron sections were prepared from pancreata frozen in OCT™ medium and fixed in ice-cold acetone for 10 min. Endogenous peroxidase activity was quenched in 3% H**_2_**O**_2_** and 0.1% sodium azide for 1 h. After treating sections with blocking reagent (Perkin Elmer) supplemented with 3% donkey serum plus 1% normal mouse serum, sections were incubated overnight with a goat anti-mouse NKp46 antibody (R&D Systems), followed by peroxidase-conjugated donkey anti-goat IgG (Jackson ImmunoResearch) for one hour and then TMR tyramide (Perkin Elmer) for 7 min. Tissue sections were then incubated overnight with guinea pig anti- mouse insulin (Invitrogen), followed by AlexaFluor 647-conjugated goat anti-guinea pig IgG (Invitrogen). Fluorescent staining patterns were detected and acquired by serial imaging on a Zeiss LSM 5 Pascal confocal microscope.

### Adoptive transfer

CD4^+^ T cells were isolated by using an AutoMacs (Miltenyi Biotec), and 5×10^6^ purified CD4^+^ T cells were transferred into immunodeficient hosts. To remove any possible contamination of CD8^+^ T cells, recipients received 200 µg of a depleting anti-CD8 mAb (53.6.7) weekly for the duration of the experiment. All recipients were checked for lymphocyte depletion at the end of the treatment by flow cytometry. An initial 500 µg of anti-NK1.1 (PK136) was given on day −1 prior to cell transfer and 100 µg weekly for the duration of the experiment. Likewise, controls were given mouse IgG2a (BioXCell).

### Disease Onset

Mice were monitored for onset of disease, considered as the first reading of ≥16 mM blood glucose, followed by two more consecutive readings of ≥16 mM blood glucose. Treated mice received either 200 µg anti-NK1.1 or a control mouse IgG2a weekly from weeks 3 to 10 weeks of age. Experimental groups were matched with littermate controls, i.e., one anti-NK1.1-treated recipient was matched with a control mouse IgG2a-treated littermate. Ten different litters were observed over a 1-year period for spontaneous onset of diabetes.

## Results

### NK cells are present in the NOD pancreas

**Figure 1:**
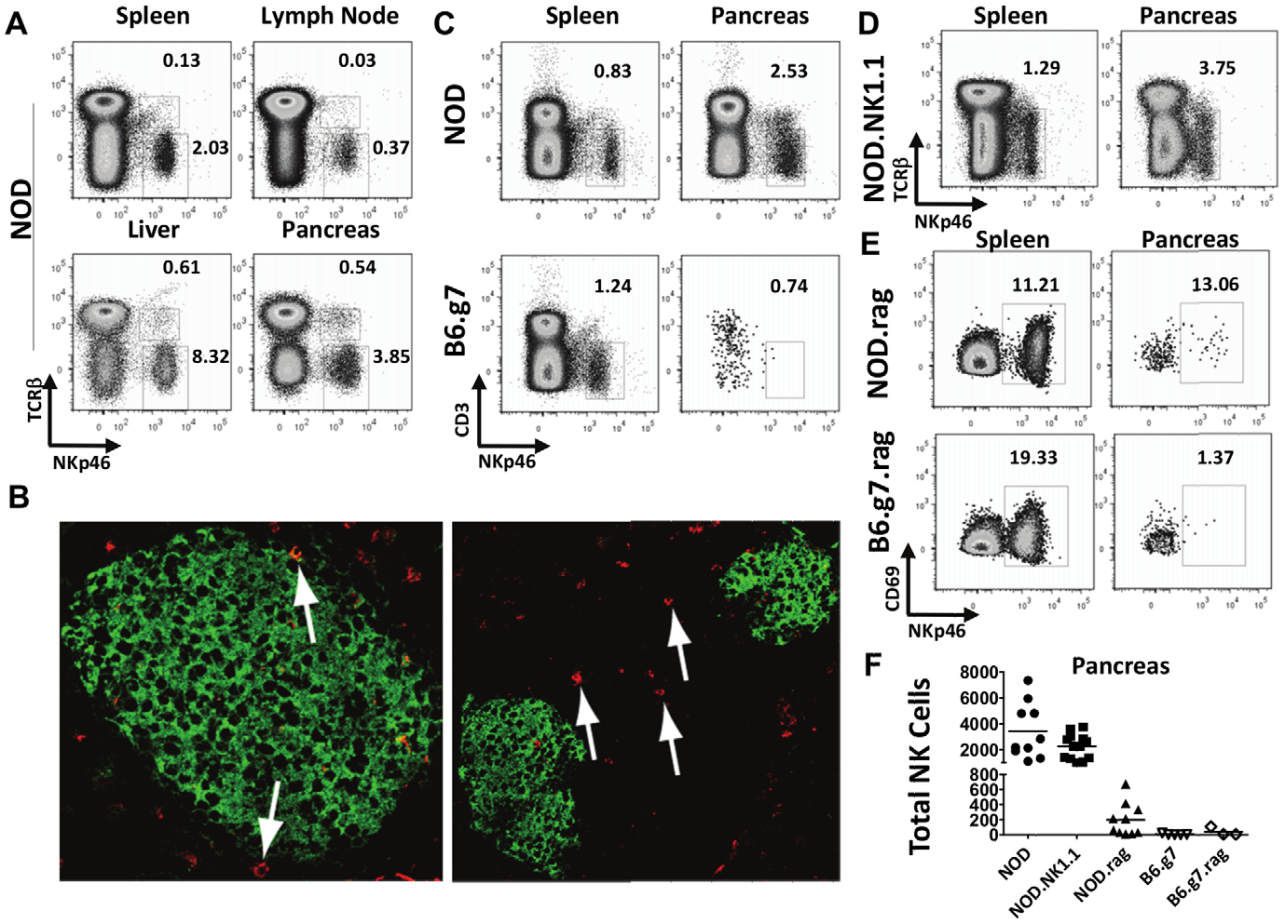
NK cells reside in the pancreas. A) Flow cytometric analysis of leukocytes isolated from enzyme-digested tissues of NOD mice. Plots are of CD45^+^ cells and numbers are percentages of CD45^+^ cells. Example of one of six similar experiments. B) Using immunohistochemistry, NK cells (red) were found in the surrounding peri-islet infiltrates (left panel 40×) and in infiltrated islets (right panel 20×). Insulin was stained green to define islets. Example of sections of pancreas from three different NOD mice, evaluating a minimum of 10 slides per mouse. C) NK cells were more numerous in NOD pancreas compared to B6.g7 pancreas– dots were enlarged to allow visualization of NK cells due to low number of events obtained from B6.g7 mice. D) NOD.NK1.1 pancreata contain similar frequencies of NK cells as NOD mice. E) *Rag2^−/−^* NOD mice have low numbers of NK cells in their pancreas, whereas *Rag1^−/−^* B6.g7 mice have very few, almost undetectable numbers of NK cells in the pancreas. F) Absolute numbers of NK cells recovered from the pancreas of individual mice from noted strains. Example from one of six similar experiments, each with a minimum of three mice per strain is shown.

NK cells have been found previously in the infiltrates of tumors and autoimmune inflammatory sites [5], [19]. NK cells were found in the pancreas of NOD mice, as demonstrated by the detection of NKp46^+^ TCRβ^−^ infiltrates by flow cytometry (Figure 1a). The percentage of NK cells consistently fell in between the frequency of NK cells present in liver and the spleen when all tissues were treated in a similar manner. Also, a unique population of NKp46^+^ TCRβ^+^ T cells was distinctly evident in the liver and pancreas (Figure 1a). The NK cells were found both in the islets and the surrounding acinar tissues (Figure 1b), in accordance with other recent findings [18]. In the non-autoimmune B6.g7 strain with the same MHC as NOD mice, NK cells were rarely found in the pancreas (Fig. 1c). NK1.1-congenic and wild-type NOD mice had equivalent frequencies of NK cells in the pancreas (Figure 1d). In some animals, NK cells were found in small numbers even in *Rag2^−/−^* NOD mice, but were extremely rare in the pancreas of Rag-deficient B6.g7 mice as detected by flow cytometry (Figure 1e), but were below our levels of detection by immunohistochemistry (data not shown). The absolute number of NK cells in the pancreas of NOD and NOD.NK1.1 mice were similar, and significantly more than in *Rag2^−/−^* NOD, B6.g7, and *Rag1^−/−^* B6.g7 mice (Figure 1f). Thus, disease-state and non-MHC genes play a role in the trafficking of NK cells into the pancreas.

### Pancreatic NK cell are proliferating and have an activated surface phenotype

**Figure 2:**
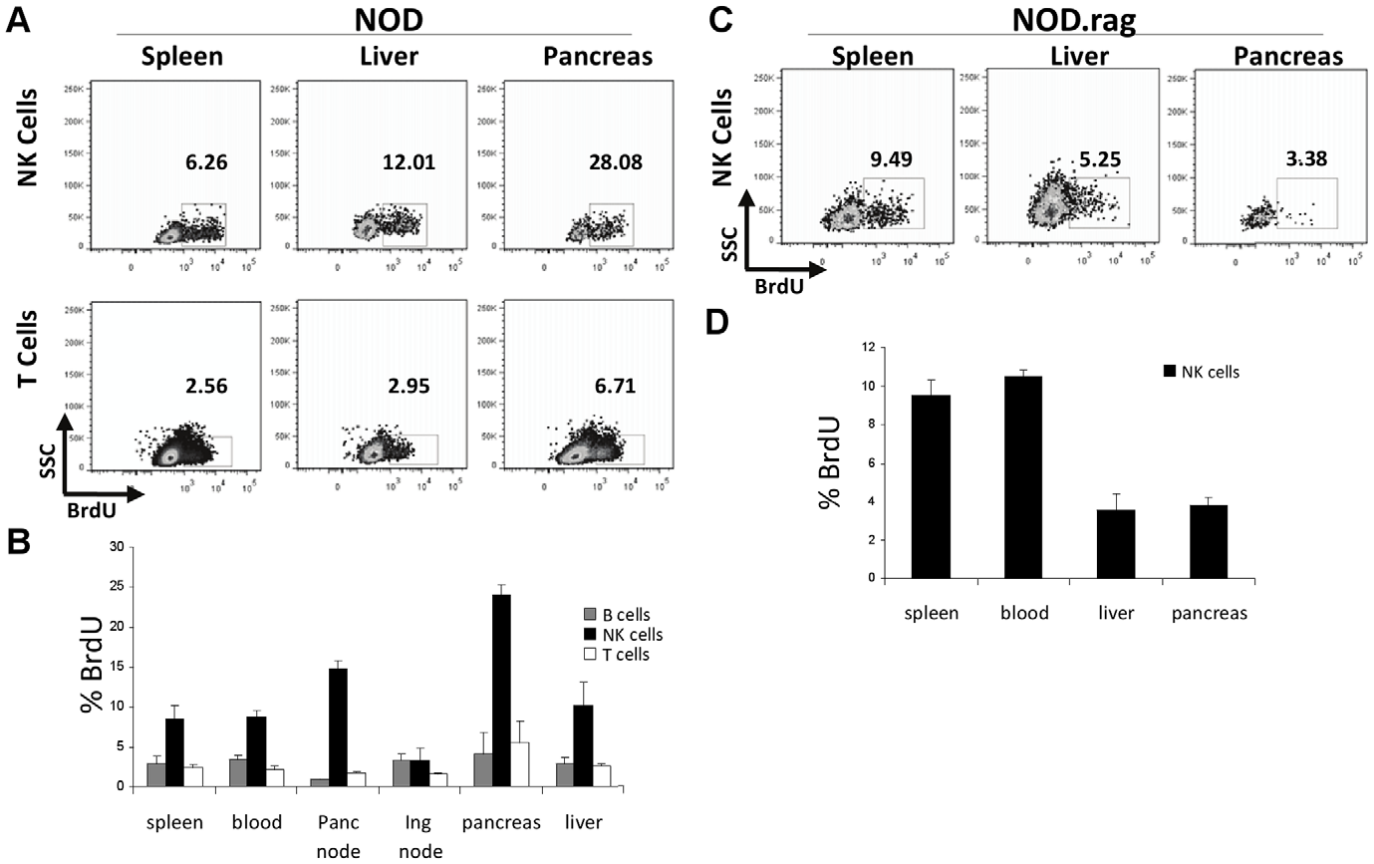
NK cells within the pancreas proliferate at a higher rate than NK cells in the periphery. Plots shown are gated on NKp46^+^ TCRβ^−^ lymphocytes day 3 after daily injections of 200 µg BrdU. A) NK cells from NOD mice incorporate BrdU at a higher rate than T cells in the same tissue. B) A higher frequency of NK cells in the pancreas are proliferating compared to NK cells in the spleen of NOD mice, relative to the same comparison of T cells in the pancreas and spleen. Bar graph of three NOD mice in one experiment C) The higher proliferation of pancreatic NK cells in NOD mice is dependent on the presence of adaptive immunity as pancreatic NK cells in *Rag2^−/−^* NOD mice did not incorporate more BrdU than splenic or liver NK cells. D) Bar graph of three *Rag2^−/−^* NOD mice. In some cases mice were pooled to obtain sufficient numbers of NK cells. One representative experiment of three independent experiments is shown.

We compared the phenotypes of NK cells isolated from the pancreas of 8 week-old NOD mice to NK cells in the spleen and liver. A higher proportion of NK cells isolated from the pancreas incorporated BrdU in comparison to NK cells found in the spleen and liver and to T cells in the same tissues (Figure 2a). While a higher percentage of NK cells incorporated BrdU compared to other lymphocyte populations throughout all tissues, a higher frequency of NK cells in the pancreas and pancreatic lymph nodes incorporated BrdU relative to other lymphocytes when compared to NK cells from other tissues (Figure 2b). This was specific to the disease state as intra-pancreatic NK cells from *Rag2^−/−^*NOD mice did not display this increased proliferation (Figure 2c and 2d). These data suggest that the NK cells from the diseased pancreas proliferate at higher rates than NK cells found in other tissues.

**Figure 3:**
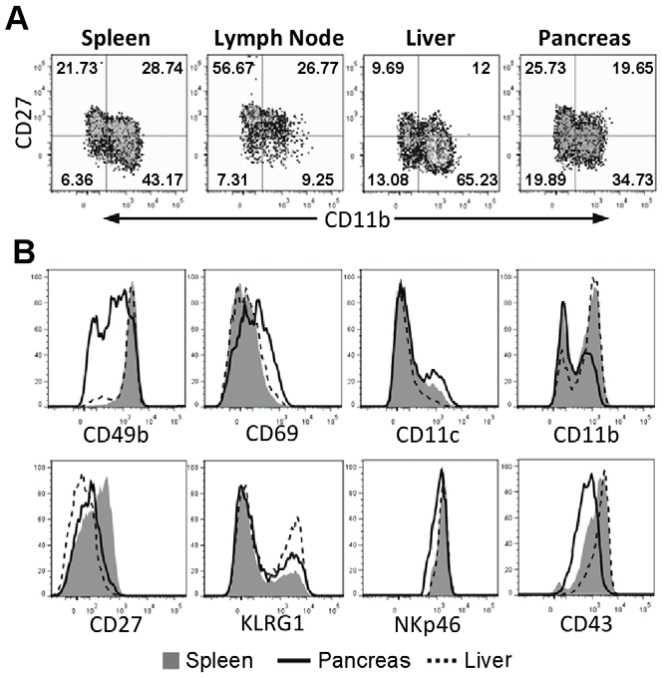
Pancreatic NK cells have an activated phenotype. A) NK cells pooled from five 8-week-old NOD mice were assayed for maturational status using CD11b and CD27. Both the pancreas and liver had more CD11b^lo^CD27^lo^ NK cells than in the spleen. B) Histograms of CD11b and CD27 (gated on CD3-, NKp46^+^ cells) demonstrate a lower mean fluorescence intensity of these markers on pancreatic NK cells relative to splenic NK cells. CD11c, CD69, and KLRG1 were increased on pancreatic NK cells. Lower expression of CD49b, CD43, and NKp46 on pancreatic NK cells was also indicative of activation. Example from one of five similar experiments is shown.

The maturation states determined by CD11b and CD27 staining [20] differed on pancreatic NK cells and splenic NK cells (Figure 3a) from NOD mice as determined by comparing the relative percentages of NK cells expressing these markers. However, the cell surface density of both of these markers (as determined by mean florescence intensity) was lower on pancreatic NK cells relative to splenic NK cells (Figure 3b). Pancreatic NK cells from NOD mice had slightly higher levels of CD69 and a higher percentage of NK cells expressing CD11c, and lower expression levels of CD49b, CD43, and NKp46 than on splenic NK cells (Figure 3b). This difference in phenotype was not general to tissue-residing NK cells as NK cells isolated from liver were more similar to splenic NK cells, with exception that NK cells in liver displayed lower amounts of CD27 and a higher frequency of KLRG1^+^ NK cells than splenic NK cells (Fig. 3b). These data indicate that pancreatic NK cells are activated and proliferating more than NK cells in other tissues of NOD mice.

### Pancreatic and liver NK cells have impaired ex vivo responses

**Figure 4:**
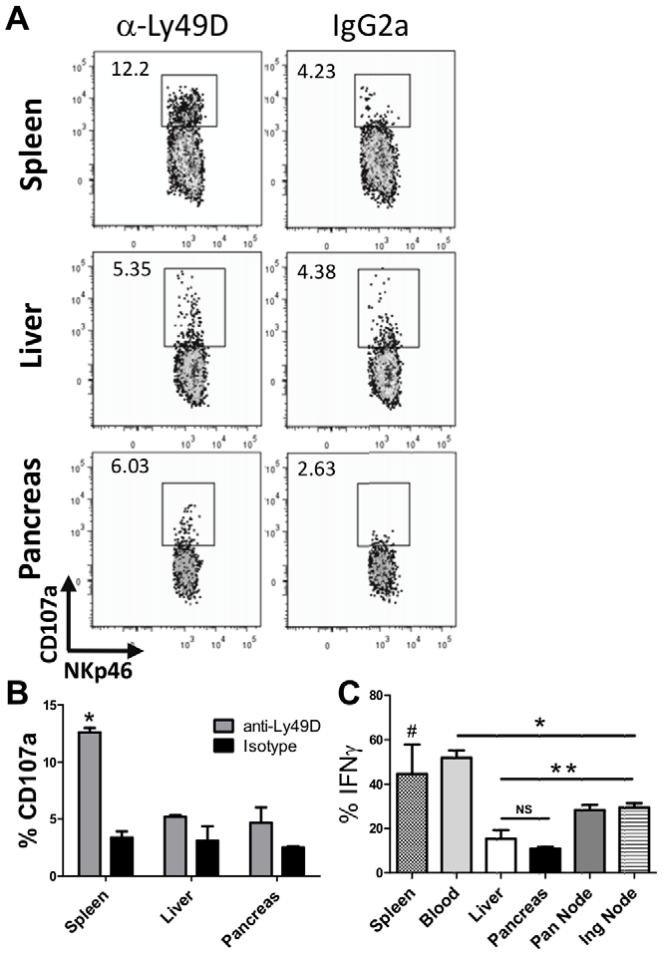
*Ex vivo* cytolytic potential and cytokine secretion are lower in pancreatic and liver NK cells. A) NK cells pooled from five 8-week-old NOD mice were stimulated for four hours with plate-bound anti-Ly49D in the presence of an antibody to CD107a to mark cytolytic granule release. B) Summary of results from 3 experiments. * p<0.05 versus all other groups (unpaired T test). C) NK cells pooled from 8-week-old NOD mice were stimulated with IL-12+IL-18 in the presence brefeldin A for four hours and then stained for intracellular IFNγ. Results are pooled from 4 independent experiments. * The frequency of NK cells producing IFNγ in blood was significantly higher than NK cells in pancreatic (Pan) or inguinal (Ing) lymph nodes (*p*<0.005) and in the liver and pancreas (*p*<0.0001), **% of NK cells expressing IFNγ in both nodes was higher than in the pancreas (*p*<0.001), # % of NK cells expressing IFNγ in the spleen was significantly higher than in pancreas (*p* = 0.04). NS, there was not a significant difference between the percentage of NK cells expressing IFNγ in the liver and pancreas according to an unpaired T test.

Primed and activated NK cells have more robust responses compared to resting NK cells [2], [4]. Using plate-bound antibodies to the activating NK cell receptor Ly49D and CD107a as a marker of granule release, we examined the cytolytic potential of NK cells. Although the frequency of NK cells expressing Ly49D was equivalent in spleen, liver, and pancreas, pancreatic NK cells had a decreased ability to degranulate relative to those isolated from the spleen. NK cells isolated from the liver were similar to pancreatic NK cells (Figure 4a and 4b). To determine if this hyporesponsiveness was restricted to Ly49D receptor-induced activation, we stimulated NK cells with a combination of IL-12 and IL-18. In these experiments, NK cells from the pancreas and liver showed a lower cytokine response relative to NK cells from peripheral blood and secondary lymphoid tissues (Figure 4c). NK cells were isolated from all tissues using similar techniques, suggesting that NK cells isolated from organ tissues compared to secondary lymphoid tissues are refractory in their *ex vivo* responses.

### In vivo responses by pancreatic NK cells are intact

**Figure 5:**
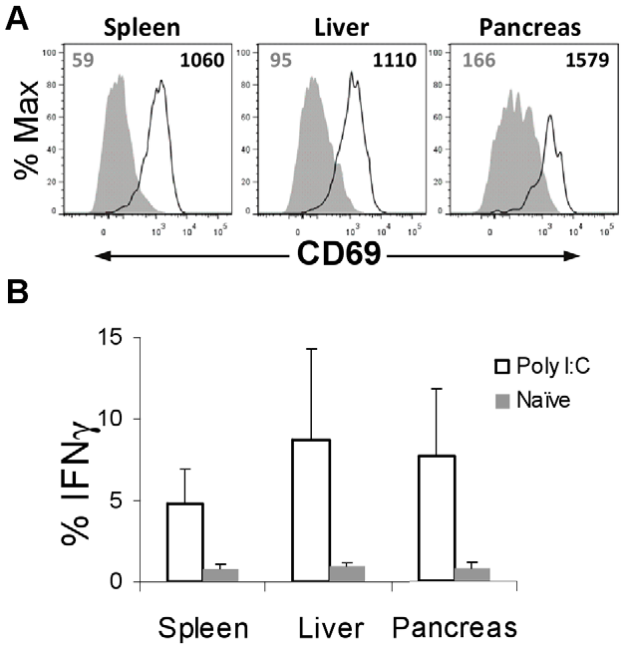
*In vivo* activation of pancreatic NK cells is intact. A) CD69 expression on NK cells from NOD mice injected with 200 µg poly I:C (solid line) versus control NOD mice injected with PBS (filled histogram). Colored numbers matching the respective histograms indicate median fluorescence intensity. One representative experiment of three independent experiments is shown. B) NK cells harvested from the mice in (A) were immediately stained *ex vivo* (without any *in vitro* stimulation) for intracellular IFNγ. Results were combined from 3 independent experiments.

Given the possibility that isolation from organ tissues might compromise NK cell function, we analyzed NK cell responses to the TLR3 agonist, polyinosinic-polycytidylic acid (poly I:C), *in vivo*. The NK cell response induced by poly I:C is indirect via antigen-presenting cells, and thus requires cell-cell and receptor-mediated interactions [21]. Increased expression of CD69 was similar in NK cells from the spleen, liver, and pancreas (Figure 5a). Likewise, immediate *ex vivo* intracellular staining of IFNγ (no *in vitro* stimulation) demonstrated no difference in the NK cell response from these three tissues (Figure 5b). Thus, the response of pancreatic and liver NK cells *in vivo* to poly I:C is similar to those found in the spleen.

### Pancreatic NK cells have no influence on intra-islet DC maturation

**Figure 6:**
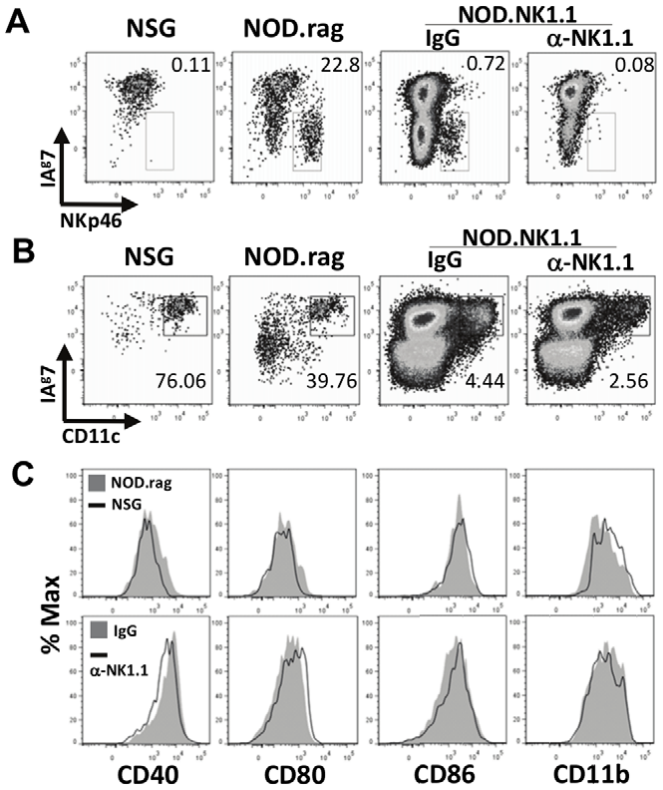
NK cells do not influence intra-islet DC maturation in NOD mice. A) NK cells were not detected in islets from NSG mice (first panel on left) or NOD.NK1.1 mice treated with anti-NK1.1 (far right panel), but were found in islets from *Rag2^−/−^* NOD mice and NOD.NK1.1 mice treated for 8 weeks with isotype-matched control IgG. Plots are of CD45^+^ cells isolated from islets of 10 week-old mice and numbers represent percentages of total CD45^+^ cells. B) DCs were detected in islets of NSG and *Rag2^−/−^* NOD mice, and in the islets of NOD.NK1.1 mice treated with isotype-matched control IgG or anti-NK1.1 (α-NK1.1). Plots are of CD45^+^ cells isolated from islets of 10 week-old mice and numbers represent percentages of total CD45^+^ cells. C) NK cells in *Rag2^−/−^* NOD mice do not promote DC maturation relative to the NK cell-deficient NSG mice (top row) as there was no difference in maturation marker expression on DC in *Rag2^−/−^* NOD and NSG mice. NOD.NK1.1 mice treated with anti-NK1.1 or an isotype-matched control IgG have DCs of similar maturation status (bottom row). One representative experiment of three independent experiments is shown.

In addition to cytokine production and killing, NK cells can have effects on the immune response through cognate interactions with dendritic cells (DC) [22]. We examined the influence of NK cells on intra-islet DC maturation as determined by expression of CD40, CD11b, CD86, and CD80 [23]. We used the disease-free NSG model, NOD.Cg-*Prkdc^scid^ Il2rg^tm1Sug^*/JicTac mice that lack NK cells (Figure 6a), to define the ‘resting’ intra-islet DC phenotype. CD45^+^CD11c^+^I-A^g7+^ cells were found within isolated islets from all strains of mice with or without the presence of NK cells (Figure 6b). The small number of NK cells found in the islets from *Rag2^−/−^* NOD mice did not promote any further DC maturation when compared to the NSG DCs (Figure 6c-top row). Moreover, DCs from anti-NK1.1-treated NOD.NK1.1 mice versus control mouse IgG2a-treated NOD.NK1.1 mice demonstrated no difference in DC maturation (Fig. 6c-bottom row). Thus, the presence of NK cells in the pancreas does not influence the maturation status of DCs.

### NK cells are not required for disease

**Figure 7:**
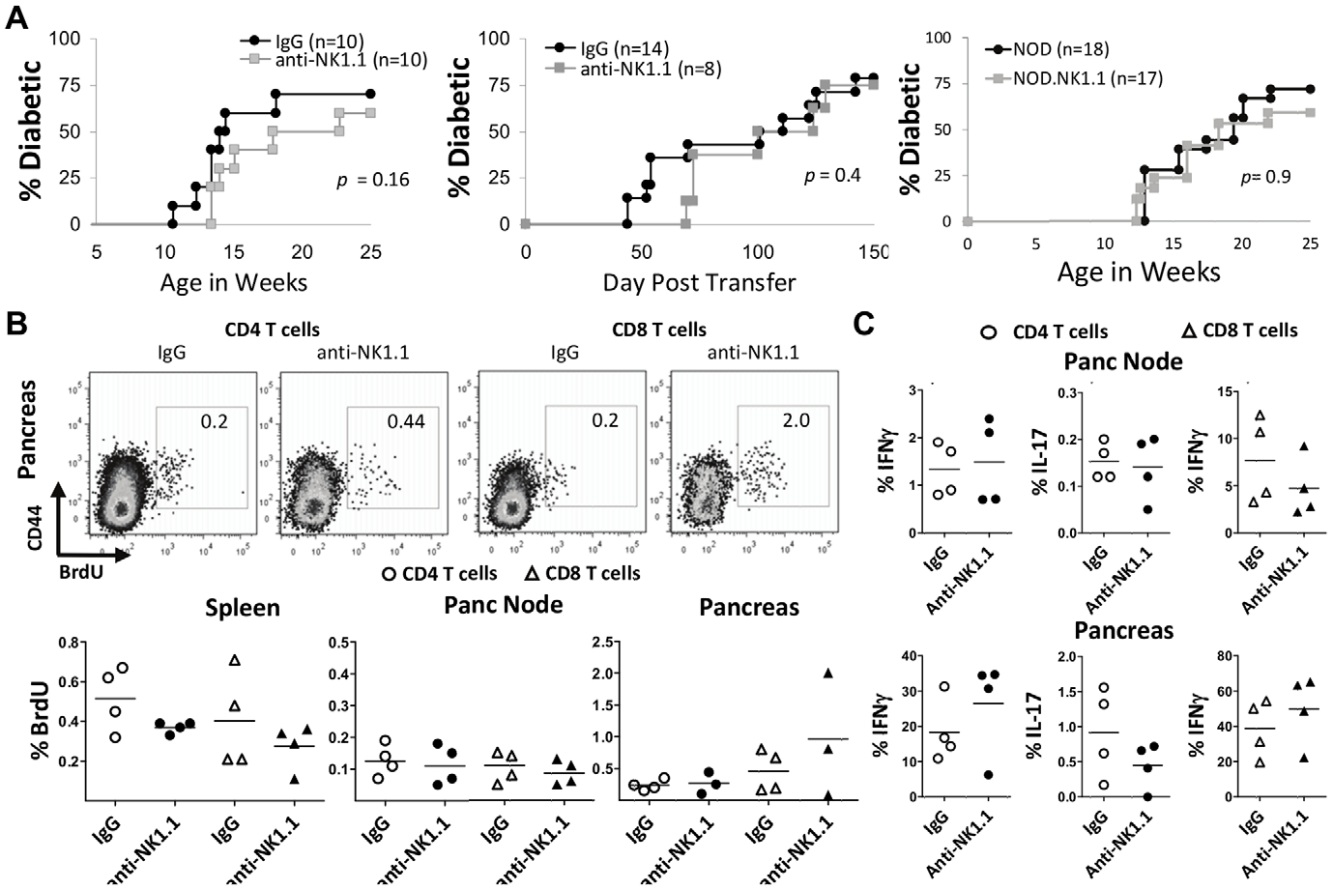
NK cells are not required for the progression to diabetes. A) The left panel shows the rate of spontaneous diabetes in NOD.NK1.1 littermates that received either anti-NK1.1 or an isotype-matched control IgG from 3 through 10 weeks of age (10 litters). In the middle panel, purified CD4^+^ T cells from diabetic NOD mice were transferred into *Rag2^−/−^* NOD.NK1.1 mice and treated with anti-NK1.1 or an isotype-matched control IgG. Both groups in the adoptive transfer experiments also received anti-CD8 to ensure exclusively CD4^+^ T cell-mediated disease. The far right panel shows the incidence of disease in unmanipulated NOD mice and NOD.NK1.1 mice in our colony. P values were calculated using the Mann-Whitney U test. B) Five hundred micrograms of BrdU was in injected intravenously and mice were sacrificed one hour later. Flow cytometric plots of cells isolated from the pancreas from one representative experiment are shown. Graphs are cumulative of 3 independent experiments involving littermates. C) NOD.NK1.1 mice were sacrificed after 10 weeks after receiving noted treatment regimens. Isolated leukocytes from the pancreatic lymph node or pancreas with stimulation with PMA and ionomycin for 3 hours in the presence of GolgiStop and assayed for intracellular IFNγ or IL-17 production by CD4^+^ T cells or CD8^+^ T cells, as indicated. Graphs are cumulative of 3 independent experiments involving littermates. No significant difference between groups was demonstrated in any parameter assayed.

Islet inflammation has been detected as early as four weeks of age in NOD mice [24]. Thus, NK cell depletion in NK1.1-congenic NOD mice was started when the pups reached 14 days-old to eliminate NK cells before any initiation of disease; NK cell depletion was efficient even after 8 weeks of therapy (Figure 6A). Full recovery of peripheral NK cells levels was not seen until a minimum of 14 days later, at 12 weeks of age. Disease onset was similar in both NK cell-depleted and control IgG2a-treated groups (Figure 7a). Adoptive transfer of purified CD4^+^ T cell from recent-onset diabetic NOD mice into *Rag2^−/−^* NOD.NK1.1 recipients, also treated with anti-CD8 to eliminate any contaminating CD8^+^ T cells, demonstrated similar results. In this model, the elimination of endogenous recipient NK cells had little influence on disease (Figure 7a). The loss of NK cells impacted the disease rate by slightly delaying the onset of transferred disease, but not significantly. The penetrance of CD4^+^ T cell-induced disease was similar with or without NK cells, with 75% of recipients becoming diabetic by 150 days. The results were similar to the spontaneous wildtype model in that the onset rate was slightly delayed in the absence of NK cells (and NKT cells, which are also depleted by anti-NK1.1 treatment), but the final disease penetrance was similar. Thus, NK cells are not required for disease progression and diabetes onset in NOD.NK1.1 mice.

Further detailed analysis of T cells from 10 week-old mice also did not display any overt differences between NK cell-depleted mice and NK cell-replete mice. BrdU analysis demonstrated no differences in CD4^+^ and CD8^+^ T cell proliferation in the pancreas, draining pancreatic lymph node, and spleen of mice depleted of NK cells compared to controls (7b). Cytokine responses, detected by intracellular staining of *ex vivo* re-stimulated T cells, demonstrated no difference in cytokine production by CD4^+^ or CD8^+^ T cells in the pancreatic nodes (Figure 7c). Similar results were noted when examining pancreatic infiltrating T cells (Figure 7c). Thus, there was no significant difference in the T cell profiles of anti-NK1.1-treated NOD.NK1.1 mice compared to isotype control-treated mice.

## Discussion

In this study, we examined the contribution of NK cells to spontaneous diabetes in NOD and NOD.NK1.1 mice. NK cells from NOD mice do not express a marker that can be targeted by currently available antibodies for depletion and therefore this study required the use of the NK1.1-congenic NOD strain to allow for *in vivo* targeted depletion of NK cells. Using NKp46 as a marker for NK cells in NOD mice, we found that NK cells were found in comparable numbers in the diseased pancreata of NOD mice and NOD.NK1.1 mice.

The presence of significant numbers of NK cells in the pancreas is specific to the NOD mouse and disease state. Few (or in some experiments no) NK cells were found in the pancreas of B6.g7 mice as detected by flow cytometry; however, these levels were consistently lower than in *Rag2^−/−^* NOD mice, which had much fewer numbers of NK cells in the pancreas compared to diseased NOD mice. These results differ from another study in which they found significant numbers of NK cells in the pancreas of B6.g7 mice [18]. The method of cell isolation from the pancreas might be responsible for this difference as we perfused our mice to remove any circulating NK cells from the blood prior to tissue harvest; it is unknown if the previous study followed a similar protocol. We also did not detect NK cells in the pancreas of *Rag2^−/−^* NOD mice and B6.g7 by immunohistochemistry. Collectively, data from experiments evaluating *Rag2^−/−^* and wild-type NOD mice suggest that the NOD pancreas harbors a unique environment for NK cells, but the infiltration of significant numbers of NK cells requires the presence of adaptive immunity.

Pancreatic NK cells in NOD and NOD.NK1.1 mice have an activated surface phenotype, but results from our *ex vivo* stimulation assays suggest that pancreatic NK cells are somewhat hyporesponsive as measured by their capacity to degranulate or produce IFNγ, consistent with prior finding [18]. Yet, because the pancreatic NK cells were similar in their responsiveness to liver NK cells in NOD mice, this hyporesponsiveness is not specific to pancreatic NK cells, but might be due to the methods used to isolate NK cells from solid organs or the unique microenvironment of tissues. Consistent with this interpretation, we have also noted a lower responsiveness of liver NK cells compared to splenic NK cells in C57BL/6 mice (our unpublished observations). In contrast, the *in vivo* response to poly I:C by liver and pancreatic NK cells was equal to that of splenic NK cells, suggesting that indeed these pancreatic NK cells are capable of responding quickly, similar to NK cells from the spleen.

Dendritic cell maturation is important to T cell activation and autoimmune disease progression [23]. DCs from *Rag2^−/−^* NOD mice displayed the same maturational phenotype as DCs from the NK cell-deficient NOG strain mice. Thus, at steady-state NK cells did not promote DC maturation in the absence of an adaptive immune system in NOD mice. Moreover, the activation status of DCs in NOD mice was not affected by the depletion of NK cells. Therefore, NK cells are not required for pancreatic DC maturation or activation in NOD mice. Thus, when using DCs as sentinels of disease status [23], the absence of NK cells doesn't appear to inhibit the progression of inflammation in the islets.

The early deletion of NK cells in NOD.NK1.1 mice by depleting antibody treatment was used to determine the role of NK cells in disease initiation or their contribution as effectors. The adoptive transfer of only CD4^+^ T cells from diabetic NOD mice into Rag-deficient NOD hosts bypasses the need to prime and activate pathogenic CD4^+^ T cells and excludes participation of CD8^+^ T cells in induction of disease. Additionally, the time period to disease onset in this adoptively transferred CD4^+^ T cell model is similar to that of spontaneous disease in NOD mice. By depleting NK cells in the Rag-deficient NOD hosts, we used this sensitive *in vivo* assay to demonstrate that NK cells have no role in the effector phase of disease in this model. Thus, NK cells do not mediate islet destruction. Likewise, when NK cells were depleted in the model of spontaneous disease in intact NOD mice, no differences in BrdU incorporation by CD4^+^ T cells or CD8^+^ T cells were demonstrated. These data also support the finding that depletion of NK cells had no significant impact on the onset of spontaneous disease in NOD mice.

Targeting of either NKG2D or NKp46 in NOD mice prevents the onset of diabetes, but our results here suggest that these outcomes are not due to the targeting of NK cells. Indeed, NK cells can kill islets *in vitro*, and islets express ligands for the activating receptors NKG2D and NKp46 [16], [17]. NKG2D is expressed by activated T cells and was suggested as the target for anti-NKG2D therapy [16]. The study that targeted the NKp46 pathway by treating mice with a NKp46-Fc fusion protein did not directly implicate NK cells by addressing the efficacy of this therapy in the absence of NK cells in NOD mice, an important consideration given that NKp46 is expressed on some γδ T cells, some non-NK innate lymphoid cells, and a subset of αβ TCR-bearing T cells identified in our study. It is also possible that a receptor other than NKp46 recognizes this NKp46 ligand and that NKp46-Fc therapy prevents that interaction. With respect to this study, the NKp46-Fc therapy does not target the same cells as anti-NK1.1 therapy. Anti-NK1.1 depletes NK cells and NKT cells. NKT cells have been shown to be protective in NOD mice if their activity is induced [25]. NK1.1 depletion does not target NKp46^+^ NK1.1^−^ cells in the intestinal lumen that produce IL-22, which is important for gut homeostasis [26]. As noted above, IFNγ production by activated NK cells is protective from diabetes. The loss of NKp46 does appear to affect homeostasis of NK cells in the gut and their ability to produce IFNγ in response to IL-12 and IL-18. Gut homeostasis is directly linked to diabetes in NOD mice as MyD88-deficient mice only develop diabetes in germ-free conditions [27]. Of note, the important NK cell activation factor IL-18 is not required for diabetes as MyD88 signaling is necessary to process functional IL-18.

A possible explanation for low NK cell function in NOD mice is that these mice have a poorly expressed *Il15* allele [28]. The rescue of NK cell activity in NOD mice by injecting IL-15 and IL-15R complexes dramatically accelerated disease onset in BDC2.5 NOD mice, suggesting that fully functional, activated NK cells may contribute to diabetes. Indeed, in our studies presented here the deletion of NK cells had modest, but not statistically significant, effects on the rate of spontaneous disease in NOD mice. However, significant delays in disease in NOD mice were demonstrated with transient depletion of either CD4^+^ or CD8^+^ T cells [29]. Thus, the recruitment and activation of NK cells in the pancreas of diseased mice appears to be a consequence of the disease pathogenesis and not a required driver of disease. In some instances, these activated pancreatic NK cells might contribute enough to the inflammatory milieu to promote disease progression, which might explain why primed CD4^+^ T cells take a little longer to promote disease in the absence of NK cells and why there is a slight delay in the spontaneous development of diabetes in NOD mice. Our results do not support a direct role of NK cells in islet destruction or a significant role for NK cells in the priming of pathogenic T cells, but future studies might explore the potential role of NK cells in the recruitment of T cells into the pancreas (e.g. by production of chemokines) since the absence of NK cells during adoptive transfer demonstrated a clear delay in onset. Finally, our finding, combined with other recent studies, suggest that NK cells can play a role in diabetes in mice on the NOD background when manipulated, but that spontaneous disease ultimately does not require NK cells.
